# The Prevalence of Neck and Back Pain among Paramedics in Poland

**DOI:** 10.3390/jcm12227060

**Published:** 2023-11-13

**Authors:** Aleksandra Bryndal, Sebastian Glowinski, Kazimiera Hebel, Julia Grochulska, Agnieszka Grochulska

**Affiliations:** 1 Institute of Health Sciences, Pomeranian University in Slupsk, Westerplatte 64, 76-200 Slupsk, Poland; sebastian.glowinski@upsl.edu.pl (S.G.); kazimiera.hebel@upsl.edu.pl (K.H.); agnieszka.grochulska@upsl.edu.pl (A.G.); 2 Institute of Physical Culture and Health, State Higher School of Vocational Education in Koszalin, Leśna 1, 75-582 Koszalin, Poland; 3 Medical Department, Poznan University of Medical Sciences, Fredry 10, 61-701 Poznan, Poland; juliagrochulska4@gmail.com

**Keywords:** paramedics, neck pain, low back pain, Neck Disability Index, Oswestry Disability Index, medical staff

## Abstract

Neck Pain (NP) and low back pain (LBP) are ubiquitous musculoskeletal conditions and some of the major causes of disability worldwide. The aim of the study was to assess the incidence and risk factors of back pain among paramedics and their correlation with the nature of work, anthropometric features and the level of recreational physical activity. A group of 201 individuals (39 females (19.40%); 162 males (80.60%)), licensed to practice as paramedics in Poland completed a questionnaire containing the author’s interview as well as the Neck Disability Index (NDI) questionnaire and the Revised Oswestry Disability Index (ODI). Among the examined paramedics, 92% of the subjects reported the presence of back pain in different parts of the spine (36% C; 17% Th; and 85% LBP). The pain intensity, determined by VAS, was on average 4.26 (SD 1.77). The level of disability, assessed by NDI, was 7.67 (SD 5.73) on average, while the ODI was 7.51 (5.90). Work-related spinal strain has a major impact on the intensity and incidence of spinal pain. Spinal pain in paramedics mainly occurs in the lower back.

## 1. Introduction

Back pain is one of the most common health complaints, encountered by approximately 60–80% of people at least once in their lifetime [1]. Back pain is one of the main causes of mental and physical discomfort, which impedes functioning in daily life and lowers self-esteem and quality of life [2,3,4,5]. It causes absenteeism from work and is the most common cause of inability to work as well as one of the main factors leading to physical disability [6,7,8,9,10,11]. From the point of view of occupational medicine, depending on whether back pain is an industrial disease or not, the level of exposure to it is related to bending the torso or the lifting and carrying of loads [12]. The disease affects specific occupational groups, among which healthcare workers are one of those most prone to it [13].

Numerous studies around the world, including Poland, have shown that many healthcare workers are exposed to a high risk of musculoskeletal diseases, especially spine pain. The lumbar and cervical parts of the spine are mainly exposed to pain [14,15,16,17,18]. Paramedics both worldwide and in Poland are at high risk of developing back pain due to the nature of the services provided; encountering stressful conditions; physically exhausting activities, such as patient care; poor working conditions; and prolonged standing [15,16,17,18].

Pain in the lumbar spine is the most common musculoskeletal disorder in the workplace [19]. It is estimated that 37% of lower back pain complaints worldwide are occupational in nature [20]. Much scientific evidence indicates that cervical spine pain is related to occupational factors, including physical work demands, and psychosocial and organizational factors. It has been shown that the main risk factors are uncomfortable body posture, heavy physical work, and repetitive and precise work. The occurrence of pain in the cervical spine is influenced by the reduction in the number of employees, the economic situation and the quality of life of these people [21,22].

Ignoring pain from the musculoskeletal system as a symptom translates into serious health consequences, ranging from discomfort to reduced quality of life as well as injuries and disability. This is accompanied by a threat to the material existence of the worker and their family, rejection by loved ones, a sense of being a burden to others and depression [23]. In light of these data, it seems necessary to make a proper diagnosis of the problem in order to assess its magnitude as well as the discomfort felt, and the effect it has on health professionals, in order to take effective preventive and corrective measures [13,24].

The aim of the study was to assess the incidence and risk factors of back pain among paramedics and their correlation with the nature of work, anthropometric features and the level of recreational physical activity.

## 2. Materials and Methods

### 2.1. Participants

The study group consisted of 201 individuals, including 39 females (19.40%) and 162 (80.60%) males. All subjects were active professionals with a paramedic license and completed the questionnaire regarding back pain and their work anonymously. The data were collected between January 2023 and June 2023.

### 2.2. Selection Criteria

Participants in the study were 18 years of age or older, professionally active and licensed. Exclusion criteria from the study were as follows: age less than 18 years, people after a spine injury, previous spine surgery, existing deformities of the spine and/or limbs, pregnant women due to static changes in the musculoskeletal system caused by pregnancy and may therefore result in this spine pain.

### 2.3. Instruments

The study was conducted based on a questionnaire containing the author’s interview, the Neck Disability Index (NDI) in the Polish version [25] and the Revised Oswestry Disability Index (ODI) in the Polish version [26]. The form with the author’s interview included questions regarding the characteristics of current and past pain. The questions concerned the possible presence and characteristics of pain in the cervical, thoracic or lumbosacral spine. Each respondent specified the location of any pain, its duration, the continuity of symptoms, and presumed cause(s) and positions that exacerbate these complaints. The questionnaire with the author’s interview also included questions on work characteristics. Questions about the profession of a paramedic concerned the dominant activities during professional work, the average number of hours worked per month, and years of professional experience. The level of physical activity, broadly understood as recreation, was also addressed. Age, body weight and height were also recorded (body mass index (BMI) was calculated). The body mass index (BMI) was calculated as weight (kg) divided by height squared (m^2^) [27,28].

Those who reported any pain also described its intensity using the Visual Analogue Scale (VAS). The participant had to mark the pain intensity from 0 (no pain at all) to 10 (the most severe pain imaginable) [23].

The cervical disability index caused by cervical spine pain was assessed using the NDI in the Polish version of the questionnaire. The reliability (0.97) and the concurrent validity of NDI Polish version in the test and in the retest were good (rs = 0.42 *p* < 0.001; rs = 0.40 *p* = 0.002, respectively). [25]. This includes 10 questions regarding pain intensity, care, lifting objects, reading, headaches, work, driving, sleeping, resting and ability to concentrate.

The disability index caused by lumbar spine pain was the Polish version of the ODI questionnaire. The reliability was 0.85, and validity of ODI Polish version was 0.88 [26]. This consists of 10 questions on pain intensity, care, lifting objects, walking, sitting, standing, sleeping, sex life, social life and travelling. Each question is graded from 0 to 5 points. In both the Neck Disability Index and Oswestry Disability Index questionnaires, each question is graded from 0 to 5 points. The aggregate score is presented on a point scale of 0–50 or as a percentage value between 0 and 100%: 0–4 (0–8%)—no disability; 5–14 (10–28%)—mild disability; 15–24 (30–48%)—moderate disability; 25–34 (50–64%)—severe disability; 35–50 (70–100%)—extreme suffering and disability.

### 2.4. Procedure

The questionnaire containing the author’s interview and the NDI [20] and ODI were sent via e-mail to respondents [26].

Prior to the opportunity to complete the questionnaires, an informed consent form was presented, thus minimizing the possibility of coercion or undue influence, and the respondent was given time to consider participation in the anonymous study. It was conveyed that the results of the study would be used in medical studies, and we suggested that responses should be honest. Questionnaires were electronically distributed to Polish public and non-public medical institutions that employ paramedics. 253 people completed the questionnaires. 22 people did not meet the inclusion criteria for the study, while another 30 people filled out the questionnaires incorrectly.

This study was approved by the Bioethics Committee at the District Medical Chambers in Gdansk (KB-14/20).

### 2.5. Statistical Analysis

All statistical calculations were carried out using the StatSoft Inc. (Palo Alto, CA, USA) statistical package (2020), STATISTICA, version 13.3. Quantitative variables were characterized by arithmetic mean, standard deviation, median, minimum and maximum value (range) and 95% CI (confidence interval). In order to determine whether a quantitative variable came from a population with a normal distribution, the following tests were used: the Shapiro–Wilk W test, Lilliefors test, Kolomogorov–Smirnov test and Jarque–Bera test. In contrast, the Leven (Brown–Forsythe) test was used to verify the hypothesis of equal variances. The significance of the differences between the two groups (unrelated variables model) was tested using Student’s *t*-test (when the variances were not homogeneous) or the Mann–Whitney U test (when the applicability conditions of Student’s t-test were not met). The significance of differences between the same variable in different structures in the absence of a normal distribution of the variable was tested using the Kruskal–Wallis test and, in the case of a statistically significant result, additionally using the post hoc test. In order to ascertain the association of strength and direction between the variables, correlation analysis was applied by calculating Pearson correlation coefficients (*). Before proceeding with the examination of the interdependencies between the variables, graphs were drawn up to illustrate the strength and direction of the relationships between the variables. This allowed for the determination of whether there were any outlier points or not. The significance level in all calculations was *p* = 0.05 and marked bold in text in the table.

## 3. Results

### 3.1. Descriptive Analysis

Table 1 presents the characteristics of the study group in terms of gender, age, height, weight, BMI, length of service, number of working hours per month and working system.

In Table 2, data on the distribution of the prevalence of spinal pain, divided into cervical, thoracic and lumbosacral spine, and the occurrence of possible radiation of these complaints to one or two limbs, are taken into account. In the question concerning the prevalence of pain at a given spinal level, subjects were allowed to give more than one answer, which means that in a given group the number of subjects and the percentage distribution may be more than 100%. The paramedics most frequently reported pain in the lumbosacral spine. In the cervical and thoracic spine, pain was most frequent centrally and less frequent with radiation. In the lumbosacral spine, at a comparable level, pain occurred centrally and with radiation to one lower limb.

Table 3 presents data on the characteristics of pain among paramedics. In the study group, 185 (92.04%) individuals reported back pain during their working life, while 16 (7.96%) reported no complaints. Most people described the intensity of their pain on the VAS scale as level 5 (moderate/strong) (63 (31.34%) respondents), and level 4 (moderate) (54 (26.87%) respondents).

Table 4 presents the frequency and nature of physical activity undertaken, i.e., broadly understood recreation. In the study group, 161 individuals (80.09%) declared that they engaged in physical activity. Most often, respondents practice moderate, medium physical activity (87 people (43.28%)), physical activity lasting 10–30 min (62 people (30.85%)) or undertake it 2 to 3 times a week (103 people (51.24%)).

In the group of paramedics with pain, the activities they indicated as generating the back pain were as follows: lifting loads—126 people (62.69%); during bending—68 people (33.83%); during twisting of the torso—51 people (25.37%); during sitting—38 people (18.91%); during standing—28 people (13.93%); and during torso overstretching—27 people (13.43%).

Among the subjects, 71 individuals declared cervical spinal pain, and 126 individuals completed the NDI questionnaire. Among the subjects, 171 individuals declared lumbosacral spinal pain, and 181 individuals completed the ODI questionnaire (Table 5).

### 3.2. Statistical Analysis

**Figure 1:**
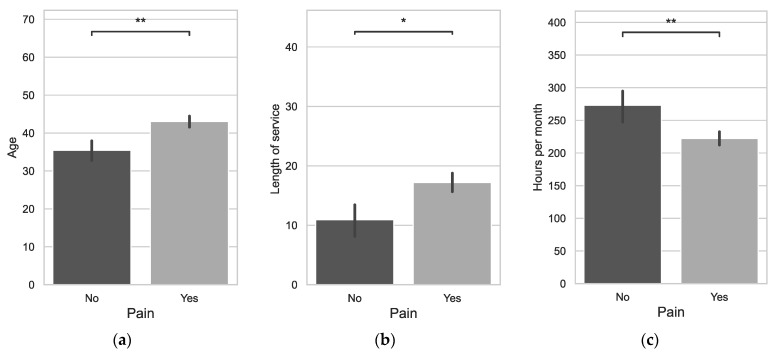
Age vs. Pain (**a**), Length of service vs. Pain (**b**), Hours per month vs. Pain (**c**); *: 1.00 × 10^−2^ < *p* ≤ 5.00 × 10^−2^; **: 1.00 × 10^−3^ < *p* ≤ 1.00 × 10^−2^.

It was observed that the average age (*p* = 0.0022) and length of service (*p* = 0.0376) were statistically significantly higher for those reporting the presence of back pain in their working life (Figure 1). It was also observed that individuals with back pain declared fewer working hours per month compared to those without pain (*p* = 0.0051).

**Figure 2:**
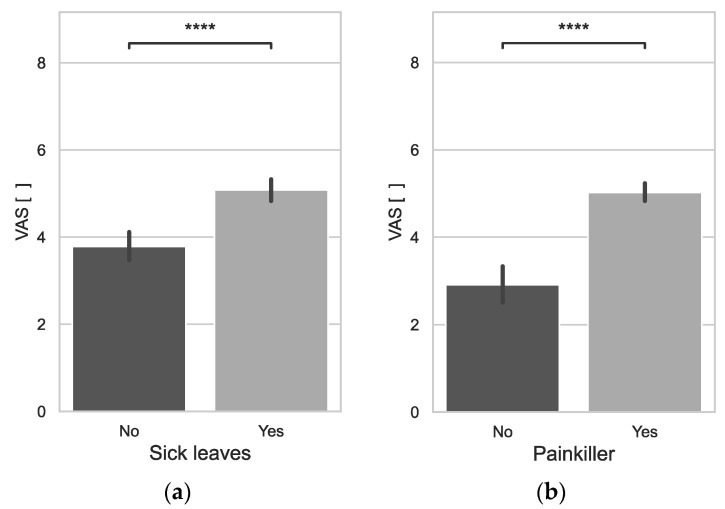
VAS Scale vs. Sick leaves (**a**), VAS Scale vs. Painkiller (**b**); ****: *p* ≤ 1.00 × 10^−4^.

The level of pain assessed using the VAS scale statistically significantly increases with age (0.2592*) and work experience (0.2599*). It was also observed that those who worked fewer hours during the month experienced higher levels of pain assessed on the VAS scale (−0.2707*). Those who had higher levels of pain on the VAS were statistically significantly more likely to take sick leave (*p* = 0.0000) and more likely to take painkillers (*p* = 0.0000) (Figure 2).

**Figure 3:**
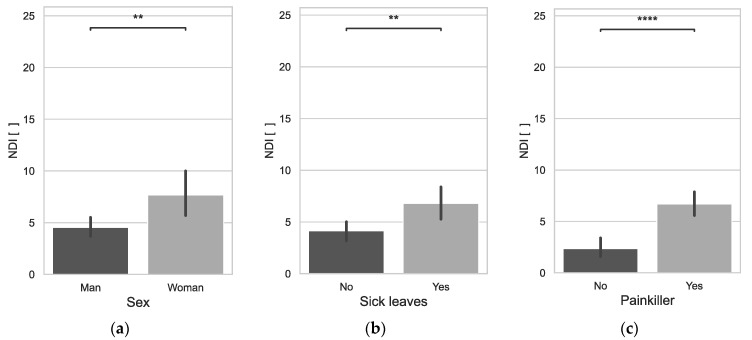
NDI vs. Sex (**a**), NDI vs. Sick leaves (**b**), NDI vs. Painkiller (**c**); **: 1.00 × 10^−3^ < *p* ≤ 1.00 × 10^−2^; ****: *p* ≤ 1.00 × 10^−4^.

The cervical disability index due to cervical spine pain assessed by means of the NDI was significantly higher in women (*p* = 0.0192) than in men (Figure 3a). It was also noted that the NDI increased significantly with age (0.1572*). Paramedics with a lower BMI had statistically significantly higher NDI values (−0.1430*). Those who had higher NDI scores were statistically significantly more likely to take sick leave (*p* = 0.0211) and more likely to take painkillers (*p* = 0.0000) (Figure 3b,c).

**Figure 4:**
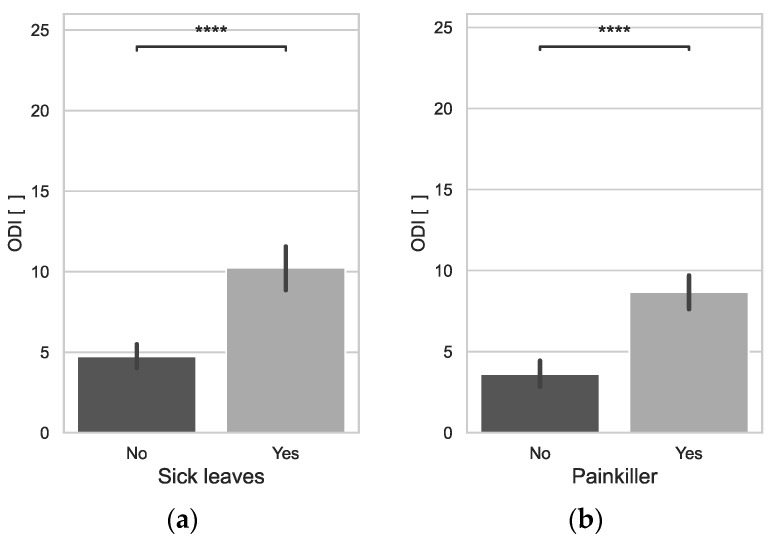
ODI vs. Sick leaves (**a**), ODI vs. Painkillers (**b**); ****: *p* ≤ 1.00 × 10^−4^.

The disability index due to lumbar spinal pain, assessed by the ODI, was significantly higher in older people (0.3933*). ODI levels increased significantly with increasing BMI (0.1791*) and work experience (0.3213*). Those who had higher ODI scores were statistically significantly more likely to take sick leave (*p* = 0.0000) and more likely to take painkillers (*p* = 0.0000) (Figure 4).

With regard to physical activity, the effects of intensity, average duration of a single workout and its frequency on the VAS, NDI and ODI levels were analyzed. The intensity of physical activity, performed as broadly understood recreational activity, significantly lead to a lower VAS level (*p* = 0.0025); lower NDI level (*p* = 0.0005) and lower ODI level (*p* = 0.0000). A longer duration of physical activity had a significant effect on lower levels of ODI (*p* = 0.0000). A higher frequency of physical activity had a significant effect on lower levels of NDI (*p* = 0.0022) and ODI (*p* = 0.0046).

## 4. Discussion

The aim of this study was to analyze the prevalence of and assess selected risk factors for back pain among paramedics. The degree of disability caused by these complaints was also assessed using the NDI scale and the ODI scale.

The main problem of the musculoskeletal system that is addressed in many studies is back pain. In various studies, back pain is the most common musculoskeletal disorder [29]. Its prevalence among healthcare professionals can impair the functioning of the healthcare system [30]. The assessment of the risk factors for these injuries is important for the development and implementation of intervention programs and the improvement of health professionals’ working conditions [31]. There are few studies addressing back pain in the cervical, thoracic and lumbosacral spine. In a study by Friedenberg et al. [32] it was found that a few studies had addressed the incidence of work-related musculoskeletal pain in relation to the incidence of shoulder, neck and arm pain. In these studies, they noted that the incidence ranged from 42% to 53% throughout the year. They observed that slightly greater limitations due to these complaints occurred in females (10%) than in males (7%). However, it should be noted that there was a large difference between the number of women (10; 0.6%) and men (1541; 99.4%), which could have influenced this result. In the same review, the annual incidence of work-related musculoskeletal pain in relation to LBP among paramedics was found to be high, ranging from 30% to 88%. In studies where the prevalence of LBP was broken down by gender, it was noted that it was in fact similar for females (42%) and males (40%) [32]. Our study analyzed the prevalence of pain among paramedics during their working lives, and it was noted that 92% of respondents reported experiencing pain in different parts of the spine (36% C; 17% Th; and 85% LBP). Such a high incidence of these complaints may be due to physical strain, mainly involving awkward body postures, lifting and carrying heavy loads. The specific job-related tasks of paramedics, such as lifting heavy objects with and without stretchers, procedures for protection mechanisms (e.g., dragging or shaking the stretcher to check its functioning), loading patients into the ambulance, and prolonged bending with torso twists during medical care affect musculoskeletal strain and the associated complaints [32]. A study by Coenen et al. [33] showed similar results regarding the association of lower back pain with activities such as lifting and carrying objects. Based on another meta-analysis, they concluded that the risk of lower back pain is associated with different levels of work-related exposures, such as the weight of an object and the frequency of lifting. This risk assessment should be the basis of workplace health policy [33,34]. In our study, we also identified lifting loads (62.69%) while bending (33.83%) and while torso twisting (25.37%) as the most common activities generating the back pain. Kuijer et al. [12] came to similar conclusions in their study. They identified a significant association of lumbosacral spinal radiculopathy with physical work, the bending/twisting of the torso, and lifting and carrying objects with simultaneous bending/twisting of the torso as risk factors for work-related back pain.

In our study, we observed that age and length of service were significantly higher for those reporting back pain. In a study by [35], the age of medical staff in general was found to be one of the risk factors for back pain, although this relationship was not very strong. As age increases, the risk of musculoskeletal disorders, especially back pain, increases too. In contrast, the findings of Tam and Yeung [36] were different as they found that younger paramedics were more likely to experience LBP. Studnek and Crawford [37], on the other hand, noted a statistically non-significant trend, i.e., that of an overall increase in the occurrence of back pain in relation to increased age.

With respect to BMI, we observed that it did not have a statistically significant effect on the occurrence of pain or its level of intensity, but we did note a higher NDI score in individuals with a lower BMI. Different results were observed by Prairie et al. [38], who concluded that being overweight was a factor contributing to back pain and suggested that overweight paramedics should lose weight to reduce load during lifting.

In our study, there was a more frequent use of sick leave for back pain in those with higher VAS, higher NDI and higher ODI. Similar results were also obtained by other authors. Aasa et al. [39] found that the frequency of sick leave due to musculoskeletal complaints of the upper and lower back ranged between 11% and 15%, and was higher in females than in males. It was additionally noted that the amount of sick leave was higher in paramedics compared to nurses and aid workers [32]. The physical requirements set for paramedics place a tremendous strain on the muscles of the back, causing muscular imbalance and stress to the musculoskeletal system.

In our study, we found that higher intensity, and longer duration and frequency of physical activity, performed as broadly understood recreation, significantly influenced a lower VAS, lower NDI and lower ODI. Other studies also confirm the beneficial effects of physical activity on the musculoskeletal system [35]. It is possible that regular exercise can help maintain proper spinal alignment by increasing the basic strength of the muscle corset, thus increasing the tolerance of loads. Improvements in the patterns of movement through sports activities area positive consequences of preventing increases in additional strain caused by abnormal posture. Increasing the strength of the spinal muscles in light of constant and repetitive spinal activity is another benefit of regular exercise. Moreover, by improving the flexibility and increasing the weight tolerance of the intervertebral discs, regular exercise will have a significant impact on improving and preventing spinal pain [35]. Pre-graduate education should include expanded classes in workload ergonomics. Healthcare workers should be covered by a preventive program and should attend regular courses on workload ergonomics. Healthcare facilities should be provided with equipment to enable work in accordance with the principles of ergonomics and should require employees to use this equipment appropriately. Educational programs and interventions in the area of risk factors and prevention of back pain should be proposed to achieve a well-functioning healthcare system.

Only paramedics from medical facilities in the northern part of Poland were included in our study, which is one of its limitations. Working conditions may vary in different healthcare regions. The findings may be useful to hospital administration and healthcare decision makers in introducing practices and methods that may reduce back strain among paramedics. There was also a difference in group size between women (39; 19.40%) and men (162; 80.60%). This could have influenced the final results. In the future, it is recommended to conduct research on a group of similar size of both sexes.

## 5. Conclusions

1. The results obtained indicate the occurrence of a health problem among paramedics—pain in various segments of the spine associated with occupational activities. Spinal strain during professional activities has a huge impact on the occurrence of such pain.
2. NDI and ODI questionnaires along with the VAS method are useful tools in the clinical assessment of patients with back pain.

## Figures and Tables

**Table 1:** Characteristics of the group of paramedics.

	Paramedics (N = 201)			
Sex	Number (% from the whole group)	Female 39 (19.40%) Male 162 (80.60%)		
Age (years)	Mean (SD) Range Me [95% CI]	42.44 (9.99) 23.0–63.0 42.0 [41.05; 43.83]	Male	43.36 (9.58) 24.0–63.0 42.0 [38.62; 10.83]
			Female	38.62 (10.83) 23.0–59.0 39.0 [35.11; 42.12]
			*p*-value	**0.0147** ^**1**^
Height (cm)	Mean (SD) Range Me [95% CI]	177.16 (8.54) 158.0–198.0 177.0 [175.98; 178.35]	Male	180.04 (6.60) 166.0–198.0 180.0 [179.02; 181.07]
			Female	165.21 (4.11) 158.0–172.0 165.0 [163.87; 166.54]
			*p*-value	**0.0000** ^**1**^
Weight (kg)	Mean (SD) Range Me [95% CI]	85.92 (16.21) 54.0–132.0 86.0 [83.66; 88.17]	Male	91.54 (14.59) 64.0–175.0 90.0 [89.27; 93.80]
			Female	65.13 (10.42) 54.0–100.0 62.0 [61.75; 68.51]
			*p*-value	**0.0000** ^**1**^
BMI	Mean (SD) Range Me [95% CI]	27.22 (4.00) 20.55–39.42 26.58 [26.66; 27.78]	Male	28.22 (4.07) 21.74–49.51 27.39 [27.59; 28.85]
			Female	23.81 (3.16) 20.55–34.60 23.14 [22.78; 24.83]
			*p*-value	**0.0000** ^**1**^
Length of service (years)	Mean (SD) Range Me [95% CI]	16.69 (10.65) 0.5–41.0 15.0 [15.21; 18.17]	Male	18.27 (10.18) 1.50–41.00 17.00 [16.69; 19.85]
			Female	10.13 (10.12) 1.00–38.00 7.00 [6.85; 13.41]
			*p*-value	**0.0000** ^**1**^
Number of working hours per month (hours)	Mean (SD) Range Me [95% CI]	226.28 (71.01) 40.00–360.00 220.0 [216.41; 236.16]	Male	237.24 (68.79) 48.00–360.00 240.0 [226.57; 247.91]
			Female	180.77 (62.10) 40.00–300.00 170.0 [160.64; 200.90]
			*p*-value	**0.0000** ^**1**^
Working system	Number (% from the entire group)	One shift	12 (5.97%)	
		Two shifts	65 (32.34%)	
		24 h duty hours	46 (22.89%)	
		Mixed	78 (38.81%)	

^1^ Mann–Whitney U test.

**Table 2:** Back pain in paramedics.

	Pain (n = 185)							
	Cervical Spine Pain			Thoracic Spine Pain		Lumbosacral Spine Pain		
Paramedics	71 (35.72%)			35 (17.41%)		171 (85.07%)		
	Central pain	Pain radiating to one limb	Pain radiating to two limbs	Central pain	Radiation	Central pain	Pain radiating to one limb	Pain radiating to two limbs
Paramedics	43 (21.39%)	25 (12.44%)	3 (1.49%)	36 (17.91%)	1 (0.50%)	84 (41.89%)	72 (35.82%)	16 (7.96%)

**Table 3:** Characteristics of pain complaints in the studied group of paramedics.

		Paramedics (n = 185)	
Intensity of pain episodes (VAS scale)	Mean (SD) Range Me [95% CI]	4.26 (1.77) 0.0–8.0 4.0 [4.01; 4.51]	
Intensity of pain episodes (VAS scale)	Number (% from the group with pain)	0 (no pain)	15 (7.46%)
		1 (very mild)	3 (1.49%)
		2 (mild)	3 (1.49%)
		3 (mild/moderate)	26 (12.94%)
		4 (moderate)	54 (26.87%)
		5 (moderate/strong)	63 (31.34%)
		6 (strong)	15 (7.46%)
		7 (strong/very strong)	13 (6.47%)
		8 (very strong)	5 (2.49%)
		9 (very strong/the strongest pain imaginable)	0 (0.00%)
		10 (the strongest pain imaginable)	0 (0.00%)
Time of the first back pain incident (years)	Number (% from the group with pain)	1 year ago	33 (16.42%)
		2–3 years ago	32 (15.92%)
		4–6 years ago	43 (21.39%)
		7–9 years ago	25 (12.44%)
		>10 years ago	52 (25.87%)
		missing data	16 (7.96%)
Number of spinal pain episodes in the past (number)	Number (% from the group with pain)		23 (11.44%)
		1–5	71 (35.32%)
		6–10	49 (24.38%)
		>11	58 (28.86%)
		missing data	0 (0.00%)
Pain characteristics and frequency	Number (% from the group with pain)	Permanent	16 (7.96%)
		Pain appears and subsides once a day	10 (4.98%)
		Pain appears and subsides once a week	47 (23.38%)
		Pain appears and subsides once a month	52 (25.97%)
		Pain appears and subsides once a year	18 (8.96%)
		Pain appeared and subsided several times in my working life	42 (20.90%)
		Pain appeared and subsided only once in my working life	0 (0.00%)
Use of sick leave	Number (% from the group with pain)	Yes	122 (60.70%)
		No	78 (38.81%)
		missing data	1 (0.50%)
Taking painkillers for back pain	Number (% from the group with pain)	Yes	130 (64.68%)
		No	69 (34.33%)
		missing data	2 (1.00%)
Use of treatment for back pain other than painkillers	Number (% from the group with pain)	I do not receive treatment	91 (45.27%)
		Medical advice	32 (15.92%)
		I receive treatment rehabilitation/physiotherapy privately	44 (21.89%)
		receive rehabilitation/physiotherapy treatment rehabilitation/physiotherapeutic treatment from the National Health Fund	16 (7.96%)
		Other ways	15 (7.46%)
Perceived limitations of mobility	Number (% from the group with pain)	I do not feel it	72 (35.82%)
		Partly	83 (41.29%)
		Limitations make my work difficult	37 (18.41%)
		Limitations prevent me from functioning independently	6 (2.99%)
		Missing data	3 (1.49%)
Use of assistance in activities at work	Number (% from the group with pain)	Assistance from another person	158 (78.61%)
		Lift	2 (1.00%)
		I do not use any other help, I do the activities on my own	38 (18.91%)
		Other assistance	0 (0.00%)
		Missing data	3 (1.49%)

**Table 4:** Physical activity in the study group of paramedics.

		Paramedics (N = 201)	
Type of physical activity undertaken	Number (% from the whole group)	I did not get engaged in physical activity	40 (19.90%)
		Mild physical activity	50 (24.88%)
		Moderate to medium physical activity	87 (43.28%)
		Intensive physical activity	24 (11.94%)
Duration of individual physical activity	Number (% from the whole group)	not applicable	41 (20.40%)
		10–30 min	62 (30.85%)
		30–50 min	54 (26.87%)
		50 min and more	44 (21.89%)
Frequency of physical activity per week	Number (% from the whole group)	1	31 (15.42%)
		2–3	103 (51.24%)
		4–5	33 (16.42%)
		6–7	4 (1.99%)

**Table 5:** ODI and NDI scores in the group of paramedics.

Paramedics		
ODI (n = 181)		
Number of participants with lumbosacral pain who completed the ODI questionnaire		
ODI	Mean (SD) Range Me	7.51 (5.73) 0.0–23.0 6.0
0–4 points (0–8%) no disability	Number (% from the group with lumbosacral pain)	79 (39.30%)
5–14 points (10–28%) mild disability		93 (46.27%)
15–24 points (30–48%) moderate disability		29 (14.43%)
25–34 points (50–64%) severe disability		0 (0.00%)
35–50 points (70–100%) extreme suffering and disability		0 (0.00%)
NDI (n = 126)		
Number of participants with cervical pain who completed the NDI questionnaire		
NDI	Mean (SD) Range Me	7.67 (5.90) 0.0–23.0 6.0
0–4 points (0–8%) no disability	Number (% from the group with cervical pain)	42 (33.33%)
5–14 points (10–28%) mild disability		65 (51.58%)
15–24 points (30–48%) moderate disability		19 (15.07%)
25–34 points (50–64%) severe disability		0 (0.00%)
35–50 points (70–100%) extreme suffering and disability		0 (0.00%)

ODI—Oswestry Disability Index; NDI—Neck Disability Index.

## Data Availability

Supporting information can be obtained from aleksandra.bryndal@upsl.edu.pl.
